# Progress in Fevers

**Published:** 1900-11-03

**Authors:** 


### PROGRESS IN FEVERS.

**The Plague.**—The infection of plague is probably communicated in various unknown ways, though sometimes there is direct inoculation of the germ into existing wounds or on to the nasal mucous membrane. Soiled clothing carries the germs, but it is not clear how they get from outside into the body, if not by the nostrils or a wound. The rat and flea theory has been actually regarded by Montenegro[1] as expressing the usual method of infection, but Nuttall has shown that there is very little real evidence for it, though it is very probable that such cases of transmission may occur.[2] While rats, monkeys, and squirrels are very susceptible to plague, horses, cows, and birds seem to be quite immune. The evidence on this point is given at length by Clemow,[3] and, indeed, it is not easy to see, even in the case of susceptible animals, how they can convey the disease to man, except through the possible medium of fleas, or when such animals bite a human being. Dogs and cats are rarely affected, and, though Simon claims to have shown that infected fleas can produce the disease in rats, it is asserted that these rat fleas do not bite human beings. How the plague is communicated from one human being to another we do not know, says G. T. Blackmore,[4] late plague medical officer for Bombay and Calcutta. He adds that the main thing is to prevent its entry into a country. Even when only a few cases have appeared the disease may remain latent, or in a very mild and unrecognisable form, for some time. After a large outbreak the same latency occurs, and fresh outbursts follow many months later. Nor can a country be pronounced free till at least twelve months have passed without a case. Thus all passengers from infected areas should be watched, and any mortality among rats should be noticed, as this gives the first indication of what is coming. Fleas and bugs should be destroyed as much as possible, rags and clothing from infected places should be disinfected, and early inoculation should be prescribed when any infection exists. In the Bombay outbreaks there was no correspondence between the temperature or season and the height of the epidemic. There was a rapid rise of mortality, then a continuance, and an equally rapid fall (Pearse[5]). Indeed, it is doubtful whether Bombay has been quite free for a single week since September, 1896. The incubation period after definite infection, such as a prick on the finger at a necropsy, is, says Clemow,[6] almost always two days, sometimes three. When infection has been contracted in the ordinary but unknown manner, the interval between exposure and the onset of symptoms is usually longer. It may, indeed, be only 24 hours, but is generally from two to seven days or more. The maximum incubation period is generally laid down as 10 or 12 days, and in longer instances the infection has probably been carried about in the clothing in the form of fomites. Zabolotony[7] holds that infection through a wound is followed by the bubonic type of disease, while the pneumonic follows infection through the nose and has a higher mortality. The blood acquires clumping powers during recovery, and immunity follows which is more certain than that given by any serum. He prefers Boux's serum to Haffkine's, and believes that recovery depends on increased phagocytosis. Treatment of 403 patients by Lustig's serum[8] in 1898 showed recoveries with the serum amounting to 38·2 per cent., while in 1,190 other patients only 19·5 got well. In another series of 610, where moribund and convalescent cases were eliminated, the corresponding figures were 39·6 per cent. and 20·2. Clemow,[9] in discussing the clinical symptoms, notices the bubonic, pneumonic, septicæmic, and enteric forms, but confesses that it is doubtful whether the infection ever enters by the intestinal canal. The temperature in hospital is usually above 103 on admission, the curve is most irregular, and in recovery the fall is usually by lysis. The appearance of the tongue is most variable. Vomiting followed by rigors and headache is an early symptom, and may be continued. The skin, the bowels, and the kidneys are inactive, and a dose of calomel and diaphoretics are valuable. There is extreme prostration with lethargy, varied by delirium or convulsions. The speech is thick and even lost for a time, and the reflexes diminish, while the pulse is very rapid and feeble, and heart failure is one of the most common causes of death in plague.

The black spots or hæmorrhages under the skin, so much spoken of in olden days, are rarely seen now, but extravasations round buboes and in the serous membranes are very common, though hæmatemesis, melena, hæmaturia, &c., are rare. Besides the form which commences with pneumonia, most patients show some lung affection of catarrhal or inflammatory type. Swollen glands forming buboes are important signs, but there is nothing constant in their characters except the marked tenderness. They may occur in the groin, the axilla, the neck, or elsewhere, and in more than one place at a time. Albuminuria is frequent, and is occasionally followed by uræmic symptoms. Altogether the symptoms and complications of the disease are very numerous, and extremely varied.

**The Glasgow Outbreak.**—The port has been under ceaseless watch since August 1899, and all ships coming even indirectly from infected places are most carefully looked after.[10] No less than six cases have been found in the Thames in the last twelve months, but in Glasgow the outbreak commenced inland, and the cases sprang from a single source. A child was taken ill on August 3rd and died on the 7th. The grandparents and several neighbours followed, and on the 25th microscopical examination of a bubo in a fresh patient showed the presence of plague. Brownlee and M'Clure remark on the existence in all the cases of headache, malaise, and vomiting, even though the fever was slight at first. Pain was generally felt in some gland or glands, and the patients' faces were marked by a peculiar dulness and apprehension. The glands were sometimes pale and sometimes red and œdematous, the tongue usually moist and the edges red. The temperature in some did not exceed 100°, while in others it was very high. Some of the cases were thought at first to be typhus or enteric. The disease had not appeared in Glasgow since 1648, though previous outbreaks were recorded in the fourteenth and sixteenth centuries, and even earlier.

In Australia the plague has not made very rapid progress. There were only 302 cases in Sydney, with 102 deaths in six months, though the population amounted to 456,000.[11] Hence we may feel some encouragement as to the probably slow spread of the disease in British communities. Cantlie[12] says the mortality among Chinese in European hospitals is about 85 per cent., among Indians 60 to 70 per cent., and among Europeans 30 to 40 per cent. Haffkine's fluid for inoculation reduces the chance of infection by 50 per cent. and the mortality by about 80 per cent. A dose of calomel followed by strychnia and stimulants is the usual course of treatment, but the various complications demand individual measures. The Yersin-Calmette curative serum has been reported on favourably in Portugal. Hewlett[13] describes the plague organism as a small bacillus, almost ovoid, often linked in pairs, staining readily by ordinary anilin dyes, but not by Gram's method. It forms a large growth in about thirty hours on blood serum or agar, while in broth with a little fat there are islands of growth on the surface and characteristic "downgrowths" from them. The diplococcus of pneumonia is distinguished by its staining by Gram's method; the plague bacillus, too, is non-motile. Guinea-pigs die within a week of inoculation, showing masses of enlarged glands and hæmorrhagic œdema round the puncture. Haffkine's fluid is made by sterilising a virulent and old growth formed in broth. Lustig's vaccine consists of the bodies of the bacilli dissolved in caustic potash and faintly acidified. Besides these fluids there are two or three kinds of serum obtained from animals by repeated injections of sterilised cultures, but the results seem inferior to those of the vaccines. The Glasgow Sanitary Department[14] call attention to the occurrence of cases of slight ailments with some rise of temperature and indefinite glandular enlargements in many places before an outbreak of plague, and insist on the importance of the early diagnosis of such instances of "Pestis minor."

[1] Med. Rec., June 16. [2] Johns Hopkins Hosp. Rep., vol. viii. Nos. 1, 2. [3] Brit. Med. Jour., May 12. [4] Lancet, June 20. [5] Lancet, June 5. [6] Lancet, May 26. [7] Brit. Med. Jour., July 28. [8] Med. Rec., Sept. 1. [9] Amer. Jour. Med. Sci., April-May. [10] Brit. Med. Jour., Sept. 8. [11] Lancet, Sept. 15. [12] Practitioner, Oct. [13] Loc. cit., p. 397. [14] Loc. cit., p. 419.

